# Assessment of cell viability of heat cured and 3D printed denture base resins on humans’ oral epithelial cells, and rats’ hepatic and myocardial cells

**DOI:** 10.1186/s12903-026-09222-5

**Published:** 2026-07-15

**Authors:** Ahmed N. Elsherbini, Nancy N. Elsherbini, Tasneem M Soliman, Mona El-Deeb

**Affiliations:** 1 Department of Prosthodontics, Faculty of Dentistry, Modern Sciences and Arts University, Cairo, Egypt; 2 Department of Prosthodontics, Faculty of Dentistry, Cairo University, Cairo, Egypt; 3 Department of Oral Biology, Faculty of Dentistry, Misr International University, Cairo, Egypt; 4 Department of Biomaterials, Faculty of Dentistry, Cairo University, Cairo, Egypt

## Abstract

**Objectives:**

Denture base materials contain unreacted monomers, which may act as bioactive compounds and affect cells and tissues in vital organs such as the liver and heart. The aim of this study was to assess the cytotoxic and cell viability effect of 3 Dimensional printed (3D) and heat cured acrylic (HCR) denture base resins on oral epithelial cells in humans and hepatic and myocardium cells in rats.

**Materials and methods:**

The Sulfo-rhodamine B (SRB) assay was used to evaluate cell viability. In 96-well plates, aliquots of 100 µL cell suspension (5 × 10^3^cells) were incubated for 24 h. Another aliquot of 100 µL media containing denture base materials, acrylic resin (heat cured acrylic resin; Acrostone) and 3D printed resin (Denture bases; iFun) in different concentrations, 0.00(control), 0.01, 0.1, 1, 10, and 100 µg/ml were used to treat the cells of human oral epithelial cells, rats’ hepatic and myocardium cells.

**Results:**

There was a concentration dependent decrease in the cell viability of human oral epithelial cells, and rats’ hepatic and myocardium cells in both heat cured acrylic resin and 3D printed resin.100ug/ml concentration caused the highest decrease in cell viability percentage. However, there was a statistical significant difference between all concentrations in the 3 cell lines. Also, morphological picture showed the heat cured resin had a more deteriorating changes in the 3 cell lines more than 3D printed resin.

**Conclusion:**

3D printed and heat cured acrylic denture base resins seemed to affect cell viability of human’s oral epithelial cells and rats’ hepatic and myocardium cells to some degree but without reaching cytotoxicity thresholds.

**Clinical relevance:**

Denture wearers wear their dentures for long period of time, and as dentures are made up of chemicals which may have a deteriorating effect on the patients’ general health. This research investigates which denture base material to be considered as the most biocompatible and safe for patients’ usage. Also, helps practitioners in choosing the more biocompatible material for their patients.

## Introduction

Several denture base materials have been used in the fabrication of prosthodontic restorations. Heat cured resin is considered the conventional and most widely used material [1]. It is composed of polymethyl methacrylate(PMMA). It can be polymerized with boiling water, light irradiation, or microwave. It has the advantage of color matching to mucosa, adaptation, and stability [2, 3]. With the advent of digital technology, 3D printed resins are now available. It has been reported that this technology enables durability, biocompatibility, esthetics, and exceptional mechanical properties [4–6].

In general, when any dental material, including denture base materials, are used intraorally they will be subject to a pool of saliva. Saliva contains many constituents such as electrolytes, proteins, enzymes, mucus, hormones, antibodies, and cells [7]. Research has shown that saliva has a degradation effect on dental materials [8]. Even though removable dentures are not worn for the whole 24 h a day, it has been demonstrated that they still undergo some degradation in the saliva leading to leakage of some of the monomer intraorally [9].

The release of the inevitable residual monomer, together with the additional monomers and chemicals released from the degraded denture base materials intraorally, can act as bioactive compounds that may eventually affect the viability of cells and tissues [10–12]. Since such chemicals can be absorbed via the sublingual route into the blood [12], they have shown to have local and systematic adverse effects [6, 9], as Monomers have shown to have several drawbacks on general health, such as skin, eyes, and mucus membrane irritation, skin rash, inflammation of mouth’s linings,, nerve damage, hepatotoxicity and fertility problems [13, 14]. Incomplete curing especially in light cured resins have shown to disturb the cell redox balance in odontoblasts in dentin [9]. It has been demonstrated that adhesive resins are cytotoxic on various cell lines, such as, human and mouse macrophages [15]. Chinese hamster fibroblast cells V79, gingival fibroblast cultures [16–18].

A survey has reported that there might be a relation between denture wearing and cardiovascular diseases, without proving the relation [19].

The aim of this study was to assess the cytotoxic and cell viability effect of the 3D printed resins versus the conventionally used heat cured acrylic denture base resins on human oral epithelial cells, hepatic and myocardium cells in rats.

The null Hypothesis there is no significant difference in cell viability and cytotoxicity between heat cured and 3D printed denture base resins across the tested cell lines.

## Materials and methods

Cell viability of Heat cured acrylic resin (HCR) (Heat cured acrylic resin; Acrostone), and 3D printed resin (3D) (Denture bases; iFun) were tested on human oral epithelial cells (OEC), and normal hepatic (BNL) and myocardium cells (H9C2) in rats. For each cell line, 5 × 10^3^ cells triplicates across three independent biological replicates were experimented.

Wax discs 1 cm diameter, 1.5 mm thickness were flasked then wax eliminated then acrylic resin (Heat cured acrylic resin; Acrostone) was packed, cured, and then deflasked. The discs were grinded to powder weighting 50µg. The 3D discs 1 cm diameter, 1.5 mm thickness were designed, and 3D printed using CAD software (exocad, exocadGMBH), then translated to a Standard Tessellation Language file “STL file”. STL file was transferred to the printer using USB flash drive. The 3D printed IFUN denture base resin was printed by a photocuring 3D printing machine (HALOT MAGE PRO 8K Resin 3D Printer, Shenzen Creality 3D Technology Co. Ltd., Shenzhen, China) with 8000 µw/cm^2^ light intensity. The discs were then grinded to powder weighting 50µg. The test was initiated directly without storage.

For blinding, the samples were delivered to the cell viability investigator labeled material 1 and material 2. All 3 cell lines were acquired from Nawah Scientific Inc.

At 37 °C humified environment with 5%(v/v) Co_2_ in streptomycin, 100 units/mL of penicillin, and 10% of fetal bovine serum activated with heat, the cells were preserved in Dulbecco’s Modified Eagle’s medium (DMEM) [22, 23].

50 µg of each denture base material were immersed in DMEM, and incubated at 37 °C for 24 h to obtain eluates. The eluates were then filtered using 0.22 μm sterile membrane filter. Concentrations ranging from 0.01 to 100 ug/ml were prepared by dilution with fresh culture medium and used for the cell viability evaluation.

The Sulfo-rhodamine B (SRB) assay was employed to assess the vitality of cells. In a 96-well plates, cell suspension (5 × 10^3^cells) in aliquots of 100 µL media were incubated for 24 h. Denture base materials HCR and 3D with the different concentrations were added to a fresh aliquot media of 100uL. This solution was used to treat the 3 cell lines. 0.00 concentration were done by adding no denture base materials to the media. After treatment, fixation of the cells was done in a media with 150 µL of 10% trichloroacetic acid (TCA), then incubated for 1 h at 4 °C. Distilled water was used to remove TCA solution, with the process repeated 5 times. Aliquots of 70 µL SRB solution (0.4% w/v) was set in a dimmed setting at room temperature for 10 min. Then the wells were cleaned with 1% acetic acid 3 times and allowed to be air dried. 150 µL of tris(hydroxymethyl)aminomethane (TRIS) was used to remove protein bound SRB and a microplate reader (Infinite F50; TECAN) was employed to find the absorbance at 540 nm [20, 21].

**Fig. 1:**
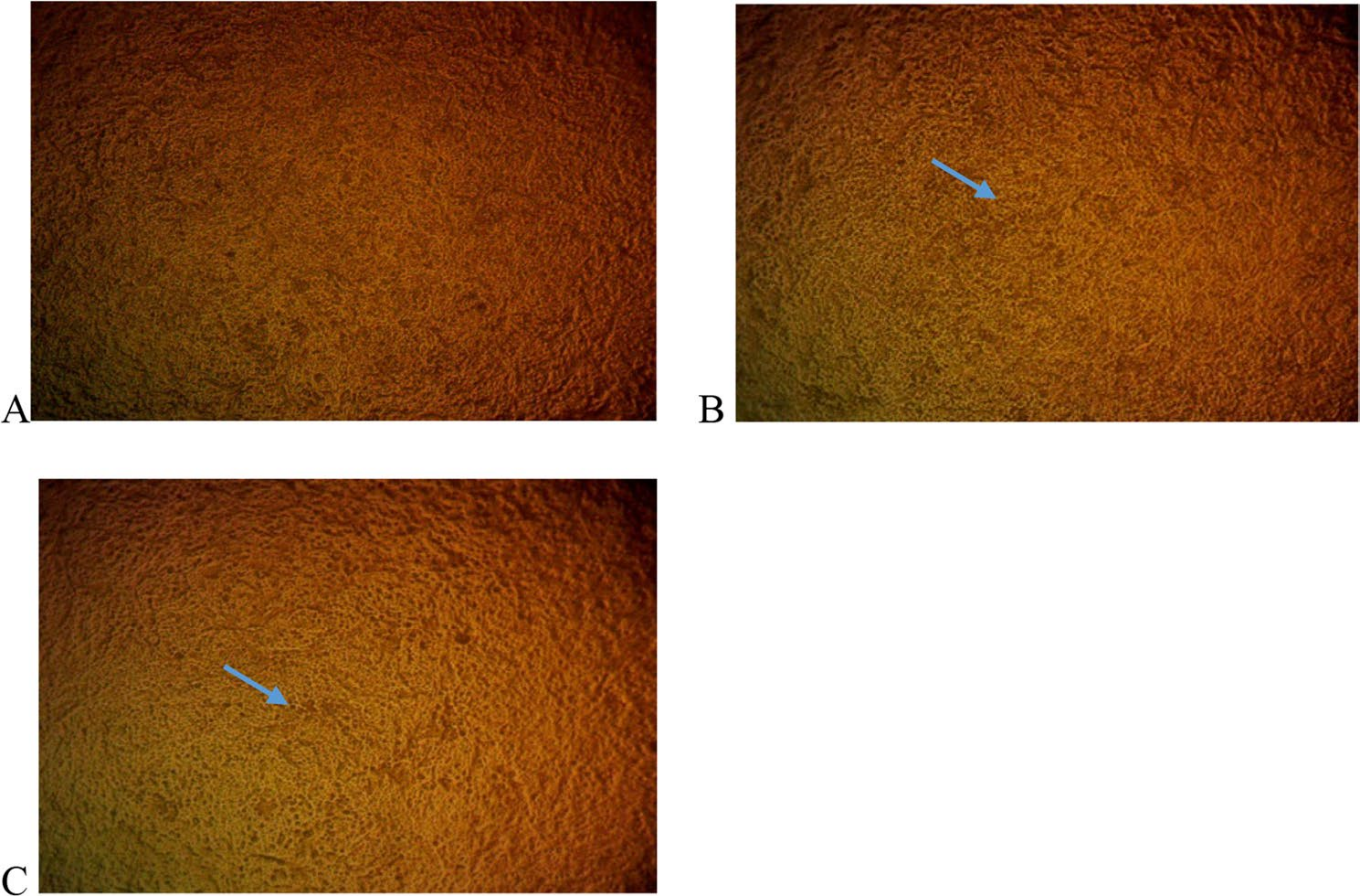
**A**: Oral epithelial cells (control, 0.00); normal, healthy morphology—ordered epithelial sheet with smooth surface texture (×40). **B**: Oral epithelial cells (3D resin, conc. 100 ug/ml); minor abnormalities—mild surface roughening, enhanced granularity, and less distinct cell boundaries (×40). **C**: Oral epithelial cells (HCR, conc. 100 ug/ml); denser, uneven texture—cell compression/clustering and distorted epithelial architecture (×40)

**Fig. 2:**
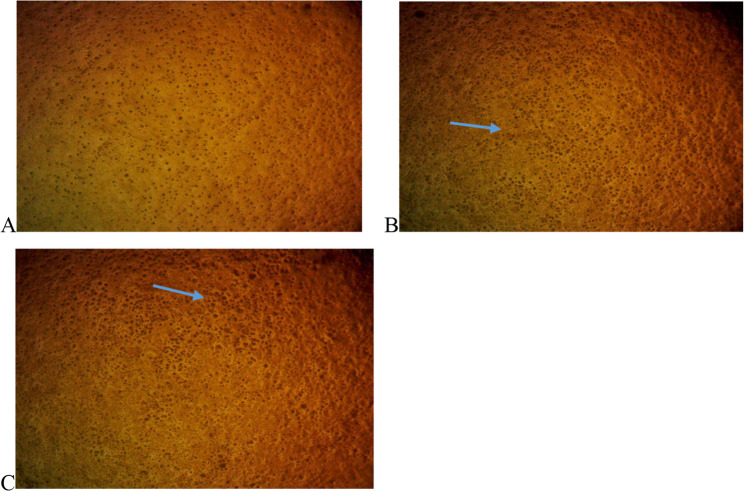
**A**: Hepatic cells (control, 0.00); uniformly distributed fine granularity with distinct cell outlines and well-preserved architecture (×40). **B**: Hepatic cells (3D resin, conc. 100 ug/ml); uneven background density, localized dark aggregate clustering, and increased granularity (×40). **C**: Hepatic cells (HCR, conc. 100 ug/ml); loss of identifiable cell outlines, dense uneven dark granular buildup, and patchy tissue distribution (×40)

**Fig. 3:**
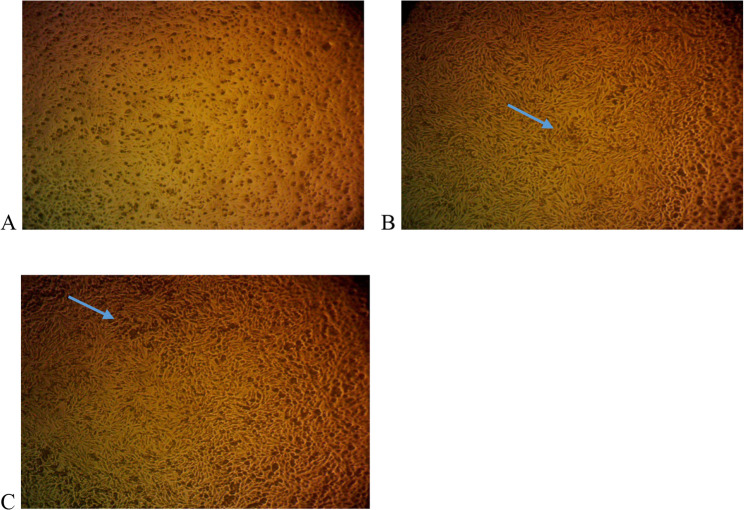
**A**: Hepatic cells (control, 0.00); network-like, healthy culture architecture with distinct alignment and directed organization (×40). **B**: Hepatic cells (3D resin, 100 ug/ml); elongated cells and overall architecture largely intact, with minor morphological changes (×40). **C**: Hepatic cells (HCR, 100 ug/ml); irregular cell shapes, less uniform orientation, and altered packing density (×40)

Microscopic images of the cells of the 3 groups were analyzed and morphological findings were documented using a microscope (CX23 binocular, Olympus) at magnification x40. Images were used to qualitatively illustrate the morphological findings among the cells in the groups, rather than quantitative measurements, therefore no scale bars were included. Figures 1(A-C), 2(A-C), 3(A-C).

Data was collected and a 1-way analysis of variance (ANOVA) test followed by Tuckey’s Post hoc test for pairwise comparison were conducted for the 2 denture base materials with different concentrations using SPSS software program (IBM SPSS Statistics, v22.0; IBM Corp) (α = 0.05).

## Results

### Qualitative results

Microscopic images under mag X40 were analyzed and morphological findings were documented, for the human oral epithelial cells tested with 3D resin and HCR shown in Table 1.

**Table 1:** Morphological findings of human oral epithelial cells under 3D printed resin and heat cured acrylic resin

Group	Morphological Findings	Interpretation	Figure
Control group	Ordered epithelial sheet with smooth surface texture, identifiable cellular outlines, and intact structural cohesion.	Represents normal, healthy epithelial morphology without cytotoxic alterations.	Figure 1A
100 ug/ml concentration of 3D-printed resin	Continuous epithelial layer with mild surface roughening, slightly increased granularity, and less distinct cell boundaries.	Indicates the onset of low-grade cytotoxic effects and early degenerative changes without major architectural disruption.	Figure 1B
100 ug/ml concentration of Heat-cured acrylic resin	Denser and uneven surface texture with cell compression/clustering and distortion of epithelial architecture. Partial disruption of intercellular connections, membrane instability, and cytoplasmic condensation observed.	Demonstrates more severe cellular damage and higher cytotoxic response compared with the 3D-printed resin group.	Figure 1C
Overall observation	Material-dependent variations in epithelial morphology were detected among the tested groups.	Suggests a material-dependent cytotoxic response.	—

Microscopic images under mag X40 were analyzed and morphological findings were documented, for rats’ hepatic cells tested with 3D resin and HCR shown in Table 2.

**Table 2:** Morphological findings of rat hepatic cells under 3D printed resin and heat cured acrylic resin

Group	Morphological Findings	Interpretation	Figure
Control group	Uniformly distributed fine granularity and distinct cellular outlines on a well-stained background with preserved architecture.	Represents normal hepatic cell morphology with intact structural organization.	Figure 2A
100 ug/ml concentration of 3D-printed acrylic resin	Cells exhibited uneven background density, localized clustering of darker aggregates, increased granularity, partially blurred cellular borders, and slight disruption of structural continuity.	Indicates early morphological alterations and mild cellular degeneration.	Figure 2B
100 ug/ml concentration of Heat-cured acrylic resin	Displayed apparent matrix collapse or compaction, loss of identifiable cell outlines, dense and uneven accumulation of dark granular material, and patchy tissue distribution.	Demonstrates severe morphological disruption and greater cytotoxic damage compared with the 3D-printed acrylic resin group.	Figure 2C

Microscopic images under mag X40 were analyzed and morphological findings were documented, for rats’ myocardium cells tested with 3D resin and HCR shown in Table 3.

**Table 3:** Morphological findings of rat myocardium cells under 3D printed resin and heat cured acrylic resin

Group / Specimen	Morphological Findings	Interpretation	Figure
Control sample	Confluent, well-organized cellular layer with distinctive spindle-shaped elongated cells arranged in interlacing bundles. Cells demonstrated a network-like architecture with distinct alignment and directed organization.	Represents healthy cardiomyocyte or cardiac fibroblast-like morphology with preserved structural integrity.	Figure 3A
100 ug/ml concentration of 3D-printed acrylic resin	Most cells retained their elongated shape and orientation with largely preserved architecture. Minor alterations included focal areas of increased cell density, moderate irregularities in alignment, and slight variations in cellular distribution. Most cells remained attached with intact cytoplasmic extensions and without widespread rounding or detachment.	Indicates mild morphological alterations with limited cytotoxic effects.	Figure 3B
100 ug/ml concentration of Heat-cured acrylic resin	More noticeable morphological alterations were observed, including less organized cellular arrangement, increased irregularity in cell shape, reduced uniformity of orientation, and altered packing density.	Demonstrates greater structural disruption and increased cellular stress compared with the 3D-printed acrylic resin group.	Figure 3C

### Quantitative cell viability results

For the cell viability test for human epithelial cells, there was a concentration dependent results, in which cell viability decreased as the concentration of denture base materials increased. In which, the mean value ± Std.dev. of cell viability % of human epithelial cells in 3D printed resin along the different concentrations were at 0.00: 100 ± 0.00, 0.01: 91.05 ± 2.84, 0.1: 88.43 ± 3.16, 1: 85.77 ± 3.92, 10: 85.30 ± 2.40, 100: 83.78 ± 1.89. F = 8.80, df = 4. The results showed statistically significant difference with *P* value < 0.000. Tuckey post hoc between different concentrations showed insignificant difference between different concentrations *P* > 0.05except for concentrations 0.01 and 0.1 *P* < 0.004, 0.01 and 10 *P* < 0.001, 0.01 and 100 *P*<0.000.

The mean value ± Std.dev. of cell viability % of human epithelial cells in Heat cured acrylic resin along the different concentrations were at 0.00: 100 ± 0.00, 0.01: 93.05 ± 2.75, 0.1: 90.34 ± 1.92, 1: 88.34 ± 2.25, 10: 82.85 ± 1.31, 100: 79.11 ± 1.12., F = 74.82, df = 4. The results showed statistically significant difference with *P* value < 0.000. Tuckey post hoc between different concentrations showed significant difference with *P* value < 0.000. however, there was a statistically insignificant difference between concentration 0.1 and 1 *P* value>0.0.064, Table 4.

**Table 4:** Mean ± Std. dv of different concentrations of 3D printed resin and heat cured acrylic resin in Human epithelial cells

Cell Line Concentration	Human Epithelial cells 3D		Human Epithelial cells HCR		*P*-value of same concentration between groups
	Mean	Std. dv	Mean	Std. dv	
0.00	100	00	100	00	**-**
0.01	91.05	2.84	93.05	2.75	0.000
0.1	88.43	3.16	90.34	1.92	.246
1	85.77	3.92	88.34	2.25	.207
10	85.30	2.40	82.85	1.31	.060
100	83.78	1.89	79.11	1.12	0.000

For the cell viability test for rats’ hepatic cells, there was a concentration dependent results, in which cell viability decreased as the concentration of denture base materials increased. The mean value ± Std.dev. of cell viability % of hepatic cells in 3D printed acrylic resin (3D) along the different concentrations were at 0.00: 100 ± 0.00, 0.01: 97.51 ± 0.20, 0.1: 95.53 ± 3.87, 1: 95.5 ± 0.66, 10: 92.28 ± 1.66, 100: 89.89 ± 1.10. F = 24.83, df = 4. The results showed statistically significant difference with *P* value < 0.000. Tuckey post hoc between different concentrations showed significant difference with *P* value < 0.000, except between concentration 0.01 and 0.1 *P-* value > 0.20, 0.01 and 1 *P-* value > 0.18, 0.1 and 1 *P-* value > 1.

The mean value ± Std.dev. of cell viability % of hepatic cells in Heat cured acrylic resin (HCR) along the different concentrations were at 0.00: 100 ± 0.00, 0.01: 94.73 ± 2.31, 0.1: 93.30 ± 0.83, 1: 90.11 ± 0.91, 10: 88.88 ± 0.50, 100: 87.98 ± 0.54. F = 51.28, df = 4. The results showed statistically significant difference with *P* value < 0.000. Tuckey post hoc between different concentrations showed significant difference with *P* value < 0.000, except between concentration 0.01 and 0.1 with *P* value < 0.009. there was a statistically insignificant difference between concentration1 and 10 *P* value > 0.211 10 and 100 *P* value > 0.531, Table 5.

**Table 5:** Mean ± Std. dv of different concentrations of 3D printed resin and heat cured acrylic resin in Rat Hepatic cells

Cell Line Concentration	Rat Hepatic cells 3D		Rat Hepatic cells HCR		*P*-value of same concentration between groups
	Mean	Std. dv	Mean	Std. dv	
0.00	100	00	100	00	**-**
0.01	97.51	0.20	94.73	2.31	0.000
0.1	95.53	3.87	93.30	0.83	.026
1	95.5	0.66	90.11	0.91	0.000
10	92.28	1.66	88.88	0.50	0.000
100	89.89	1.10	87.98	0.54	0.000

For the cell viability test for rats’ myocardium cells, there was a concentration dependent results, in which cell viability decreased as the concentration of denture base materials increased. The mean value ± Std.dev. of cell viability % of myocardium cells in 3D printed acrylic resin along the different concentrations were at 0.00: 100 ± 0.00, 0.01: 97.56 ± 3.12, 0.1: 91.16 ± 2.10, 1: 90.16 ± 2.32, 10: 86.45 ± 0.89, 100: 83.57 ± 1.67. F = 54.76 df = 4. The results showed statistically significant difference with *P* value < 0.000. Tuckey post hoc between different concentrations showed significant difference with *P* value < 0.000, except between concentration 0.1 and 1 *P*>0.862 and concentration 10 and 100 *P*> 0.0517.

The mean value ± Std.dev. of cell viability % of myocardium cells in Heat cured acrylic resin along the different concentrations were at 0.00: 100 ± 0.00, 0.01: 99.74 ± 2.24, 0.1: 95.49 ± 0.30, 1: 92.23 ± 1.10, 10: 86.82 ± 2.77, 100: 85.63 ± 2.63. F = 75.22, df = 4. The results showed statistically significant difference with *P* value < 0.000. Tuckey post hoc between different concentrations showed significant difference with *P* value < 0.000, however, there was a statistically insignificant difference between concentrations 10 and 100 with *P* value > 0.731, Table 6.

**Table 6:** Mean ± Std. dv of different concentrations of 3D printed resin and heat cured acrylic resin in Rat myocardium cells

Cell Line Concentration	Rat myocardium cells 3D		Rat myocardium cells HCR		*P*-value of same concentration between groups
	Mean	Std. dv	Mean	Std. dv	
0.00	100	00	100	00	**-**
0.01	97.56	3.12	99.74	2.24	.001
0.1	91.16	2.10	95.49	0.30	0.000
1	90.16	2.32	92.23	1.10	0.000
10	86.45	0.89	86.82	2.77	.001
100	83.57	1.67	85.63	2.63	.003

## Discussion

The null hypothesis of this study is rejected although all the results reported in this study showed that it had some effect on cell viability but not to the extent of being cytotoxic. Maybe it causes some inflammatory response that may have resulted in that but not to the degree that it is cytotoxic, as any materials to be considered cytotoxic should cause at least 30% loss of cells according ISO recommendations [17, 18]. Our results coincide with Weżgowiec et al. [22] in which it was reported that HCR and 3D caused cytotoxicity but was within the accepted threshold recommended by ISO.

Since the results of the tested materials did not exceed this threshold, it could be assumed that they are somehow safe and non-toxic, with heat cured acrylic resin causing some degree of cytotoxicity more than 3D printed resin [22, 23].

Heat cured acrylic resin caused less cell viability in the human epithelial cells when compared with 3D printed resin. In addition, the morphological pictures, showed a material-dependent cytotoxic response, with heat cures acrylic resin having a more potent, detrimental impact on epithelial integrity and 3D printed acrylic resin causing modest epithelial stress. This result coincides with Jorge et al. [3] who reported that the cytotoxicity of acrylic resins used in prosthodontic equipment can vary greatly, one of the main causes of cytotoxicity is residual monomer including methyl methacrylate (MMA), which are released gradually and impair cellular survival [3]. Compared to heat-cured acrylic resin, 3D printed resin leached less free monomer [10].

In rats’ hepatic cells, the heat cured acrylic resin also caused less cell viability and more cytotoxic effect than 3D printed acrylic resins in the human epithelial cells. The effect can be related to the influential effect of the residual monomer [6, 9]. Boran et al. [14] reported that PMMA can have a cytotoxic effect on liver cells.in humans. However, Rao et al. [24] reported that biochemical, hematological, and histopathological characteristics were examined in a PMMA toxicity test on several tissues. The liver, kidney, spleen, and subcutaneous tissues showed no changes.

In rats’ myocardium cells, the 3D printed resin and HCR showed a decrease in cell viability but within the acceptable threshold. In a survey it presented that dentures can be associated cardio vascular diseases without emphasizing the real relation between both, however in this study they didn’t indicate the material used in the fabrication of the dentures [19]. In the morphological picture, Rat myocardial cells seem to be more biologically affected by heat cured acrylic resin than by 3D printed acrylic resin.

It was advised to immerse the freshly made dentures for 24 h before to patient delivery in order to lessen the impact of free monomer leaching. Bural et al. [25] reported that the majority of free monomer leaching happens in the first 24 h.

## Conclusion

Across the tested cell types, heat-cured acrylic resin generally demonstrated a more detrimental cellular response than 3D-printed acrylic resin. Morphological observations further supported these results, showing greater evidence of cellular stress and tissue damage with heat-cured acrylic resin, while 3D-printed resin induced comparatively milder effects, including increased stress markers without overt massive cell detachment or cell death at the assessed time points. Taken together, the results suggest that 3D-printed acrylic resin exhibits more overall biocompatibility, while heat-cured acrylic resin shows comparatively reduced compatibility with epithelial, hepatic, and myocardial cells.

### Limitations

The study was performed on isolated cells in controlled laboratory conditions. Cellular responses in living organisms (in vivo) are influenced by immune reactions, metabolism, blood flow, and clearance, which are not fully represented in vitro. Cell viability was evaluated at specific time points. However, residual monomer release from the denture bases may occur over longer periods, and some toxic effects may be delayed. Although rat cells are widely used for preliminary biocompatibility and cytotoxicity assessment, however their biological response may not completely replicate human cellular response.

## Data Availability

No datasets were generated or analysed during the current study.

## Abbreviations

3D: 3 Dimensional
HCR: Heat Cured Acrylic Resin
SRB: Sulfo-rhodamine B
DMEM: Dulbecco's Modified Eagle's medium
TCA: Trichloroacetic acid
TRIS: Tris(hydroxymethyl)aminomethane
